# Supplementary material for: Curated and harmonised transcriptomics datasets of interstitial lung diseases
Source: Data Brief. 2025 Oct 14;63:112139. doi: 10.1016/j.dib.2025.112139 (PMC12581653; doi:10.1016/j.dib.2025.112139)
Supplement: Supplementary file 1 [file mmc1.zip › Supplementary_material/RNA-seq/fastQC reports/GSE138283/SRR10212986_fastqc.html]

SRR10212986.fastq.gz FastQC Report 

FastQC Report

Fri 2 Feb 2024  
SRR10212986.fastq.gz

## Summary

- Basic Statistics
- Per base sequence quality
- Per sequence quality scores
- Per base sequence content
- Per sequence GC content
- Per base N content
- Sequence Length Distribution
- Sequence Duplication Levels
- Overrepresented sequences
- Adapter Content

## Basic Statistics

| Measure | Value |
| --- | --- |
| Filename | SRR10212986.fastq.gz |
| File type | Conventional base calls |
| Encoding | Sanger / Illumina 1.9 |
| Total Sequences | 99640692 |
| Sequences flagged as poor quality | 0 |
| Sequence length | 35-76 |
| %GC | 47 |

## Per base sequence quality

## Per sequence quality scores

## Per base sequence content

## Per sequence GC content

## Per base N content

## Sequence Length Distribution

## Sequence Duplication Levels

## Overrepresented sequences

| Sequence | Count | Percentage | Possible Source |
| --- | --- | --- | --- |
| CCACAAATTATGCAGTCGAGTTTCCCACATTTGGGGAAATCGCAGGGGTCAGCACATCCGGAGTGCAATGGATA | 432014 | 0.4335718583728825 | No Hit |
| CCCACTACCACAAATTATGCAGTCGAGTTTCCCACATTTGGGGAAATCGC | 370040 | 0.37137437784956373 | No Hit |
| CCTTAGGCAACCTGGTGGTCCCCCGCTCCCGGGAGGTCACCATATTGATG | 325895 | 0.32707018935597115 | No Hit |
| CACAAATTATGCAGTCGAGTTTCCCACATTTGGGGAAATCGCAGGGGTCA | 314172 | 0.3153049157868153 | No Hit |
| CCCCACTACCACAAATTATGCAGTCGAGTTTCCCACATTTGGGGAAATCGCAGGGGTCAGCACATCCGGAGTGCA | 309454 | 0.3105699025052937 | No Hit |
| CTTCGATGTCGGCTCTTCCTATCATTGTGAAGCAGAATTCACCAAGCGTT | 254372 | 0.2552892747874533 | No Hit |
| CTGGAGTCTTGGAAGCTTGACTACCCTACGTTCTCCTACAAATGGACCTTGAGAGCTTGTTTGGAGGTTCTAGC | 215037 | 0.21581243133076594 | No Hit |
| CTTAGGCAACCTGGTGGTCCCCCGCTCCCGGGAGGTCACCATATTGATGC | 193274 | 0.19397095315235266 | No Hit |
| CTCCGTTTCCGACCTGGGCCGGTTCACCCCTCCTTAGGCAACCTGGTGGT | 171157 | 0.17177419843691974 | No Hit |
| CACTACCACAAATTATGCAGTCGAGTTTCCCACATTTGGGGAAATCGCAG | 161832 | 0.16241557214395902 | No Hit |
| GGGAGTTTGACTGGGGCGGTACACCTGTCAAACGGTAACGCAGGTGTCCT | 158213 | 0.15878352189685718 | No Hit |
| CAGGCTGGAGTGCAGTGGCTATTCACAGGCGCGATCCCACTACTGATCAG | 156396 | 0.1569599697280304 | No Hit |
| TGATGATGTGTTGTTGCCATGGTAATCCTGCTCAGTACGAGAGGAACCGC | 138040 | 0.13853777731692188 | No Hit |
| CCCCTCCTTAGGCAACCTGGTGGTCCCCCGCTCCCGGGAGGTCACCATAT | 134918 | 0.135404519270099 | No Hit |
| CCAGGCTGGAGTGCAGTGGCTATTCACAGGCGCGATCCCACTACTGATCA | 129304 | 0.1297702749796238 | No Hit |
| AGCAAATATTCAAACGAGAACTTTGAAGGCCGAAGTGGAGAAGGCTTCCATGTGAACAGCAGTTGAACATGGGTC | 123156 | 0.12360010506550878 | No Hit |
| CAGAAAAGTTACCACAGGGATAACTGGCTTGTGGCGGCCAAGCGTTCATA | 122469 | 0.12291062771824185 | No Hit |
| GTTGGATTGTTCACCCACTAATAGGGAACGTGAGCTGGGTTTAGACCGTC | 113693 | 0.11410298113947262 | No Hit |
| CCCTCCTTAGGCAACCTGGTGGTCCCCCGCTCCCGGGAGGTCACCATATT | 112691 | 0.11309736789062041 | No Hit |
| CCACTACCACAAATTATGCAGTCGAGTTTCCCACATTTGGGGAAATCGCA | 109768 | 0.11016382744511649 | No Hit |
| CTCGGATCGGCCCCGCCGGGGTCGGCCCACGGCCCTGGCGGAGCGCTGAGAAGACGGTCGAACTTGACTATCT | 105670 | 0.10605104990639767 | No Hit |
| CTCAGACCGCGTTCTCTCCCTCTCACTCCCCAATACGGAGAGAAGAACGATCATCAATGGCTGACGGCAGTTGC | 102371 | 0.10274015359106499 | No Hit |

## Adapter Content

Produced by FastQC (version 0.11.7)
